# No-Go Zones for Mariner Transposition

**DOI:** 10.1128/mBio.01690-16

**Published:** 2016-10-11

**Authors:** Brian J. Akerley

**Affiliations:** University of Mississippi Medical Center, Department of Microbiology and Immunology, Jackson, Mississippi, USA

## Abstract

The property of transposons to randomly insert into target DNA has long been exploited for generalized mutagenesis and forward genetic screens. Newer applications that monitor the relative abundance of each transposon insertion in large libraries of mutants can be used to evaluate the roles in cellular fitness of all genes of an organism, provided that transposition is in fact random across all genes. In a recent article, Kimura and colleagues identified an important exception to the latter assumption [S. Kimura, T. P. Hubbard, B. M. Davis, M. K. Waldor, mBio 7(4):e01351-16, 2016, doi:10.1128/mBio.01351-16]. They provide evidence that the Mariner transposon exhibits locus-specific site preferences in the presence of the histone-like nucleoid structuring protein H-NS. This effect was shown to bias results for important virulence loci in *Vibrio cholerae* and to result in misidentification of genes involved in growth *in vitro*. Fortunately, the bulk of this bacterium’s genome was unaffected by this bias, and recognizing the H-NS effect allows filtering to improve the accuracy of the results.

## COMMENTARY

Since their discovery as mobile elements of “selfish DNA” responsible for horizontal gene transfer and genetic variability among organisms, transposons have been recognized as powerful tools to query gene function. Their defining feature, random gene insertion, generates libraries of mutants in which the disrupted loci can be readily identified, allowing phenotypes to be correlated with mutant genotypes. The development of high-throughput DNA sequencing has allowed identification of such correlations at the whole-genome scale in large libraries of transposon mutants. A variety of related techniques to identify the sequences at the junctions of transposon insertions and the genome, methods that can generally be termed transposon insertion sequencing, or TIS (reviewed in reference 1), are applied in two basic formats. In one instance, comparison of the number of transposon insertions in each gene under a selective condition of interest to that observed under standard *in vitro* growth conditions identifies genes conditionally required for fitness. In the second, by sequencing transposon junctions in the transposon bank during or after its initial expansion on media designed for optimal propagation, investigators can infer the identities of so-called “essential genes” or “growth-promoting” genes involved in cellular survival or viability *in vitro*, based on the absence or significantly reduced density of insertions present within these genes. In the latter case, the underlying assumption that transposition is primarily random is more critical, such that a role in fitness can be ascribed to loci sustaining fewer insertions than expected.

In a recent article, Kimura and colleagues analyzed the puzzling observation that some of the loci in *Vibrio cholerae* that fail to sustain transposon insertions, or that sustain insertions at markedly reduced frequencies relative to other genes after random high-density transposon mutagenesis, are dispensable for growth *in vitro* on rich medium, as determined previously with directed knockout mutations (2). Many of the genes within these loci have been functionally characterized and have no identified roles in fitness or cellular physiology in laboratory-grown cultures. Particularly notable were genes within the *tcp* locus encoding the pilus adhesin, required during colonization of the host, and the *ctx* locus, encoding this pathogen’s primary exotoxin. TIS studies have classified these genes as required for fitness of *V. cholerae* in vitro in rich medium based on a dramatically reduced transposon insertion frequency relative to that of other genes, and yet extensive study of independently derived knockout mutants has revealed no effects of these genes on *in vitro* growth or survival.

The key to tracking down a mechanism for the discrepancy was, in part, a statistical modeling approach that these investigators have developed to examine the distribution of transposon insertions in short sliding windows of potential target sites spanning the genome, allowing a topological view of insertion frequencies (3). Applying this approach, they noted a statistically significant paucity of insertions in genes regulated by the histone-like nucleoid structuring protein H-NS, which can oligomerize along its target sites to modify DNA tertiary structure, often acting as a transcriptional silencer. H-NS typically binds to DNA rich in adenine (A) and thymine (T) nucleotides, and statistical analysis confirmed that loci that were underrepresented for transposon insertions were also disproportionately rich in AT sequences.

Kimura and colleagues provide an explanation for these observations by obtaining evidence that H-NS selectively blocks transposition at its binding sites. An important point is that the mutant library was generated by introduction of a nonreplicating plasmid carrying the Mariner transposon and its transposase gene into *V. cholerae*, followed by antibiotic selection for mutants on rich medium, a commonly used approach termed “*in vivo* transposition.” The authors postulated that during *in vivo* transposon mutagenesis, H-NS is bound to its cellular target sites and might physically interfere with the recombination of the transposon at those locations. In support of this hypothesis, a comparison of insertion densities after transposon mutagenesis of wild-type *V. cholerae* versus an H-NS knockout mutant revealed an abundance of genes that sustain more insertions when H-NS is not present. Moreover, comparison of the TIS data with the results of a recent chromatin immunoprecipitation sequencing (ChIP-Seq) experiment (4) that identified H-NS binding regions indicated that sites that bind H-NS were more likely to show more transposon insertions in the H-NS knockout strain and that the skewed distribution of insertions at AT-rich versus GC-rich DNA sites is abrogated in the absence of H-NS. H-NS also affected insertion at some sites with neutral GC content; however, these are likely to result from synthetic fitness effects of such mutations in concert with the physiological roles of H-NS.

In wild-type cells, the presence of H-NS nearly doubles the number of AT-rich sites that are preferentially underrepresented with insertions compared with the H-NS knockout strain, indicating that a relatively large set of genes is likely to be nonessential for fitness but would be incorrectly inferred to be essential based on the wild-type TIS data. H-NS has been implicated in interactions with transposons (5), but, prior to the study by Kimura and colleagues, its influence on transposition has been examined only at a limited number of target sites, and interaction of H-NS with the Mariner transposon has not been previously investigated. The marked effect of H-NS on genomic transposition site preference raises the possibility that other nucleoid interacting proteins may also influence TIS experiments.

The authors noted a potential caveat in that similar results might be obtained if H-NS inhibits expression of the transposon-encoded antibiotic resistance gene used for selection of mutants. While difficult to fully exclude, this possibility seems unlikely because transposon insertion density throughout the genome does not correlate with gene expression levels at the insertion sites, consistent with independent expression of the antibiotic resistance gene in the transposon. Moreover, at least one locus, *rfb* (encoding the O antigen of the lipopolysaccharide [LPS]), was shown to exhibit an H-NS-mediated decrease in insertion density and yet is strongly expressed. Readthrough transcription into the transposon from the *rfb* genes is expected to increase rather than decrease antibiotic resistance gene expression; therefore, transcriptional repression does not appear to be sufficient to explain the low insertion density at this site in the presence of H-NS.

Inhibition of transposition at selective sites during *in vivo* transposon mutagenesis presents several issues in interpretation of TIS experiments. The most apparent problems are that genes without insertions would not be evaluated for selective loss of insertions after exposure of the mutants to experimental conditions and that the dynamic range of mutant phenotypes would be reduced for genes with unusually low initial insertion densities. The bias imparted by H-NS is particularly troublesome in this regard as it preferentially affects AT-rich DNA, often a characteristic of horizontally transmitted virulence factors. Also, a major application of TIS has been the identification of essential or growth-promoting genes. These genes are inferred to encode cellular factors essential for life and may represent previously uncharacterized components of cellular pathways. The members of this class also represent potential antibiotic targets in microbial pathogens. As shown by Kimura and colleagues, if interference with transposition, rather than fitness defects, were to cause a lack of insertions at certain locations, then some genes would be falsely identified as essential. In addition to H-NS, several other conserved bacterial proteins participate in nucleoid structure, and there may be more genes whose roles in fitness are incorrectly identified by TIS. Understanding these factors that affect transposition will aid in filtering out such false positives.

Obstruction of transposition by H-NS or other nucleoid-interacting proteins can presumably be avoided by conducting TIS after *in vitro* transposition in which cellular factors are absent during the reaction and mutagenized DNA is then introduced into bacterial cells. This approach is practical only in naturally transformable organisms such as *Haemophilus influenzae* and *Streptococcus pneumoniae* (e.g., those that are proficient at uptake of naked DNA) (6, 7). *V. cholerae* is naturally transformable (8), so it may be possible to directly examine whether this method can be used to bypass H-NS effects. Moreover, such a comparison may allow identification of additional loci at which other concurrent protein-DNA interactions interfere with *in vivo* transposition. Nevertheless, biases may also occur during *in vitro* transposition by preferential selection of sites by the transposases themselves. The *Himar1* Mariner transposon used in many TIS studies appears to have little site preference *in vitro* other than a strict requirement for the TA dinucleotide, which is relatively abundant in the genes of most organisms; however, it is somewhat affected by DNA topology, and the possibility remains that it may have unidentified selectivity (9).

Aside from aiding in interpretation of TIS experiments, these results may have important biological implications. Prior to their modification in the laboratory for use as genetic tools, transposons adapted for their optimized spread in nature by balancing propagation, transmission, and maintenance of their genomes within those of their hosts. Regulation of transposition, often involving host regulatory factors (10), can aid in maintaining this balance; however, Mariner transposons are eukaryotic elements that appear to lack their native control mechanisms when moved into bacteria. Nevertheless, the results reported by Kimura and colleagues suggest that not all regulation is absent in this context, and it is possible that cells maintain general defenses against diverse transposons at specific loci.

The discovery of pronounced genome-scale effects of H-NS on transposition may relate to the proposed role of H-NS in tolerance of recipient cells with respect to horizontal gene transfer events. DNA with AT composition higher than that of the rest of the genome is a hallmark of recently acquired genomic islands, including virulence loci such as *CTX* and *tcp* in *V. cholerae*. H-NS is postulated to protect cells from ectopic expression of newly acquired genes (11), which might otherwise be detrimental to survival, and it is known to partially repress expression of *tcp* and *CTX* genes. Transposition into newly acquired DNA could also lead to aberrant gene expression by introduction of ectopic promoters or disruption of negative-control elements, leading to inappropriate expression of horizontally acquired genes. Therefore, inhibition of transposition may allow *V. cholerae* to retain the potential benefits of gene transfer and to avoid deleterious mutations or inappropriate expression levels that may be caused by mutagenesis. Likewise, Mariner transposons may have evolved to steer clear of insertion locations that would otherwise markedly interfere with survival of their carrier organisms. How this eukaryotic transposon might recognize bacterial H-NS to discern these disadvantageous sites is unclear. Mariner transposons are influenced by chromatin structure, and it is possible that they can recognize conserved features of genomic DNA structure generated by bacterial nucleoid proteins. Further study will be required to evaluate the potential adaptive role of inhibition of transposition by H-NS binding and whether it is generalizable to other classes of transposons.
